# Evaluating the clinical utility of early exome sequencing in diverse pediatric outpatient populations in the North Carolina Clinical Genomic Evaluation of Next-generation Exome Sequencing (NCGENES) 2 study: a randomized controlled trial

**DOI:** 10.1186/s13063-021-05341-2

**Published:** 2021-06-14

**Authors:** Brooke S. Staley, Laura V. Milko, Margaret Waltz, Ida Griesemer, Lonna Mollison, Tracey L. Grant, Laura Farnan, Myra Roche, Angelo Navas, Alexandra Lightfoot, Ann Katherine M. Foreman, Julianne M. O’Daniel, Suzanne C. O’Neill, Feng-Chang Lin, Tamara S. Roman, Alicia Brandt, Bradford C. Powell, Christine Rini, Jonathan S. Berg, Jeannette T. Bensen

**Affiliations:** 1 Department of Epidemiology, Gillings School of Global Public Health, University of North Carolina at Chapel Hill, Campus Box #7295, Chapel Hill, NC 27599-7295 USA; 2 Department of Genetics, University of North Carolina at Chapel Hill, Chapel Hill, NC 27599 USA; 3 Department of Social Medicine, University of North Carolina at Chapel Hill, Chapel Hill, NC USA; 4 Department of Heath Behavior, Gillings School of Global Public Health, University of North Carolina at Chapel Hill, Chapel Hill, NC 27599 USA; 5 Cecil G. Sheps Center for Health Services Research, University of North Carolina at Chapel Hill, Chapel Hill, NC USA; 6 Lineberger Comprehensive Cancer Center, University of North Carolina, Chapel Hill, NC 27599 USA; 7 Department of Pediatrics, University of North Carolina at Chapel Hill School of Medicine, Chapel Hill, NC 27599 USA; 8 Center for Health Promotion and Disease Prevention, University of North Carolina at Chapel Hill, Chapel Hill, NC USA; 9 Department of Oncology, Georgetown University, Washington, DC 20007 USA; 10 Department of Biostatistics, University of North Carolina at Chapel Hill, Chapel Hill, NC 27599 USA; 11 Department of Medical Social Sciences, Northwestern University Feinberg School of Medicine, Chicago, IL USA; 12 Robert H. Lurie Comprehensive Cancer Center, Northwestern University, Chicago, IL USA

**Keywords:** Precision medicine, Sequencing, Under-represented populations, Clinical trial, Genetic disease, Diagnostic odyssey, Community engagement, Patient education, Question prompt list, ELSI

## Abstract

**Background:**

Exome sequencing (ES) has probable utility for shortening the diagnostic odyssey of children with suspected genetic disorders. This report describes the design and methods of a study evaluating the potential of ES as a routine clinical tool for pediatric patients who have suspected genetic conditions and who are in the early stages of the diagnostic odyssey.

**Methods:**

The North Carolina Clinical Genomic Evaluation by Next-generation Exome Sequencing (NCGENES) 2 study is an interdisciplinary, multi-site Phase III randomized controlled trial of two interventions: educational pre-visit preparation (PVP) and offer of first-line ES. In this full-factorial design, parent-child dyads are randomly assigned to one of four study arms (PVP + usual care, ES + usual care, PVP + ES + usual care, or usual care alone) in equal proportions. Participants are recruited from Pediatric Genetics or Neurology outpatient clinics in three North Carolina healthcare facilities. Eligible pediatric participants are < 16 years old and have a first visit to a participating clinic, a suspected genetic condition, and an eligible parent/guardian to attend the clinic visit and complete study measures. The study oversamples participants from underserved and under-represented populations. Participants assigned to the PVP arms receive an educational booklet and question prompt list before clinical interactions. Randomization to offer of first-line ES is revealed after a child’s clinic visit. Parents complete measures at baseline, pre-clinic, post-clinic, and two follow-up timepoints. Study clinicians provide phenotypic data and complete measures after the clinic visit and after returning results. Reportable study-related research ES results are confirmed in a CLIA-certified clinical laboratory. Results are disclosed to the parent by the clinical team. A community consultation team contributed to the development of study materials and study implementation methods and remains engaged in the project.

**Discussion:**

NCGENES 2 will contribute valuable knowledge concerning technical, clinical, psychosocial, and health economic issues associated with using early diagnostic ES to shorten the diagnostic odyssey of pediatric patients with likely genetic conditions. Results will inform efforts to engage diverse populations in genomic medicine research and generate evidence that can inform policy, practice, and future research related to the utility of first-line diagnostic ES in health care.

**Trial registration:**

ClinicalTrials.govNCT03548779. Registered on June 07, 2018.

**Supplementary Information:**

The online version contains supplementary material available at 10.1186/s13063-021-05341-2.

## Trial Status

Patient recruitment began on 8/28/2018 under Version 1 of IRB protocol #17-0816 and is anticipated to end on 05/31/21.

## Background

For patients with a suspected genetic condition, the “diagnostic odyssey” can be a period of considerable uncertainty characterized by multiple clinical interactions and potentially distressing procedures aimed at identifying the underlying etiology of the patient’s symptoms [1, 2]. Often commencing at birth or early in childhood and sometimes continuing well into adulthood, the diagnostic odyssey can be emotionally and financially draining for patients and families. Genome-scale sequencing (GS), used to collectively refer to sequencing of the protein-coding regions (the exome) or the entire genome, is an evolving technology with demonstrated utility for identifying molecular diagnoses among people with suspected underlying genetic disorders [3–9]. Expanding clinical understanding of sequence variants, improvements in sequencing technologies, and decreasing costs are driving the clinical use of diagnostic GS, including in neonates and critically ill infants with suspected genetic disorders [10–14]. However, there is still much to learn about the potential impact of GS in diagnosing conditions with clinical presentation in childhood or later that are often genetically heterogenous and elude diagnosis through routine medical care. Thus, it is essential to evaluate the utility of GS for pediatric patients early in their diagnostic odyssey.

Important research toward this aim, including studies conducted by the Clinical Sequencing Exploratory Research (CSER) consortium [15], has established the diagnostic yield of GS and explored the feasibility of the clinical integration of sequencing technologies [15–18]. However, this body of literature also highlighted the need for additional research about optimal and equitable clinical integration of GS, such as cost-benefit tradeoffs of GS compared with traditional approaches to diagnosis in standard clinical care, short- and long-term ethical, legal, and social implications (ELSI) of genomic sequencing for families (including potential clinical and personal benefits of shortening the diagnostic odyssey), and the need to dramatically improve communication between clinicians and laboratorians and the patients, families, and communities who will receive and seek to understand their diagnostic sequencing results [18–25]. Though previous research has examined aspects of clinical GS and informed various facets of its use in clinical practice, the goal of the Clinical Sequencing Evidence-Generating Research consortium (the currently active CSER Consortium, or “CSER 2”) [19] is to provide a broader understanding through dedicated enrollment of diverse participants in various clinical settings. Successfully transitioning GS from research to standard clinical care—and convincing third-party payers to equitably adopt this technology—requires thoroughly evaluating its effects on medical decision making and clinical care, familial psychosocial dynamics, and communication processes between patients, clinicians, and laboratorians among diverse patients and clinical settings [20, 21].

The NCGENES 2 study seeks to build on the knowledge gained through the original NCGENES study [9, 22–24], which helped to identify which types of patients were most likely to benefit from application of exome sequencing (ES) [16, 22, 23, 25–30] and examine patient decision making to understand how they processed and responded to genomic information [31–37]. Attention to enrollment of a diverse study population in the NCGENES study enabled a broad range of perspectives to be elicited, but also raised potential challenges regarding enrollment and attrition of participants from populations under-represented in traditional research [38]. Given that historical exploitation and exclusion of low-resourced or non-White research participants have resulted in disparities and barriers to health care access [15, 39–41], it is vital to investigate clinical applications of diagnostic GS with more diverse cohorts to improve health equity in genetic medicine [19]. Developing optimal and equitable genomic implementation requires engaging participants from diverse social, economic, and cultural backgrounds. Partnering with a range of institutions in different geographic areas can facilitate this engagement and aid our understanding of variations in clinical barriers to implementation.

NCGENES 2 is a Phase III randomized controlled trial that will apply and extend our prior findings on how to employ diagnostic ES in routine medical care with a specific focus on understanding and addressing the needs of underserved and under-represented populations throughout the study [38]. It will address specific outcomes of first-line or early use of ES in the context of a diagnostic odyssey, an emerging area of potential impact in genomic medicine. Procedures in the NCGENES 2 study will be incorporated in the usual clinical process for recruited patients who will be having their initial visit to pediatric genetics or pediatric neurology clinics. Additionally, NCGENES 2 will employ methods to establish a more diverse cohort with respect to race/ethnicity and socioeconomic status. It will also include an intervention designed to examine the impact of helping families prepare for a clinical visit that may include ES, in light of evidence showing subgroup differences and disparities in genomic knowledge and patient engagement, with implications for patient outcomes [33].

Fundamental issues surrounding clinical use of ES will be addressed by the NCGENES 2 study, including the technical challenges of genomic data sharing and interpretation; multi-level stakeholder engagement and communication among families, patients, clinicians, and laboratorians; and the efficacy of a low-cost, evidence- and education-based intervention to increase family members’ engagement in clinical consultations. In addition, the study will investigate other overarching questions of interest about whether early ES in diverse and medically underserved children improves physicians’ diagnostic thinking, decisions about patient management, patients’ health outcomes, and overall healthcare utilization. This paper summarizes the design and methods protocol that will be employed in the NCGENES 2 study.

## Methods/design

### Study design overview

The NCGENES 2 study is led by researchers at the University of North Carolina at Chapel Hill, one of six sites participating in the CSER 2 consortium, which is a national multi-site research program jointly funded by the National Human Genome Research Institute (NHGRI), the National Cancer Institute (NCI), and the National Institute on Minority Health and Health Disparities (NIMHD). The CSER 2 consortium sites and a coordinating center use interdisciplinary, translational research to evaluate the integration of GS into the clinical care of diverse and medically underserved individuals with suspected genetic disorders by developing and sharing best practices in areas such as informed consent, informed and shared decision making, patient-reported outcomes, and return of results.

NCGENES 2 follows up, in part, on exploratory research conducted during the original NCGENES study [22], which performed ES in 643 adult and pediatric patients to evaluate its utility as a diagnostic test. In this continuation study, NCGENES 2 will be a full-factorial Phase III randomized controlled trial of two interventions: (1) pre-visit preparation (PVP) for parents/guardians of patients (versus not receiving PVP) and (2) offer of ES (versus not offered ES) (Table 1).

**Table 1:** Trial arms and interventions

Arm	Interventions
**Experimental:** ***PVP / ES + usual care***	Participants randomized to pre-visit preparation (PVP) will receive a study packet with educational materials and a question prompt list (QPL) before their clinical interactions (e.g., clinic visit or return of diagnostic/clinical test results). These participants will be instructed to review the materials, use the QPL to select questions they would like to ask at their clinic visit, and use the QPL during clinical interactions. Participants will be offered research exome sequencing (ES) in addition to their usual clinical care.
**Experimental:** ***PVP / usual care***	Participants randomized to PVP will receive a study packet with educational materials and a QPL before their clinical interactions (e.g., clinic visit or return of diagnostic/clinical test results). These participants will be instructed to review the materials, use the QPL to select questions they would like to ask at their clinic visit, and use the QPL during clinical interactions Participants will not be offered research ES but will receive usual clinical care.
**Experimental:** ***No PVP / ES + usual care***	Participants in the no PVP arm will be mailed a study packet reminding them about their upcoming clinic visit. Participants will be offered research ES in addition to their usual clinical care.
**Control:** ***No PVP / usual care***	Participants in the no PVP arm will be mailed a study packet reminding them about their upcoming clinic visit. Participants will not be offered research ES but will receive usual clinical care.

### Study interventions

**Fig. 1:**
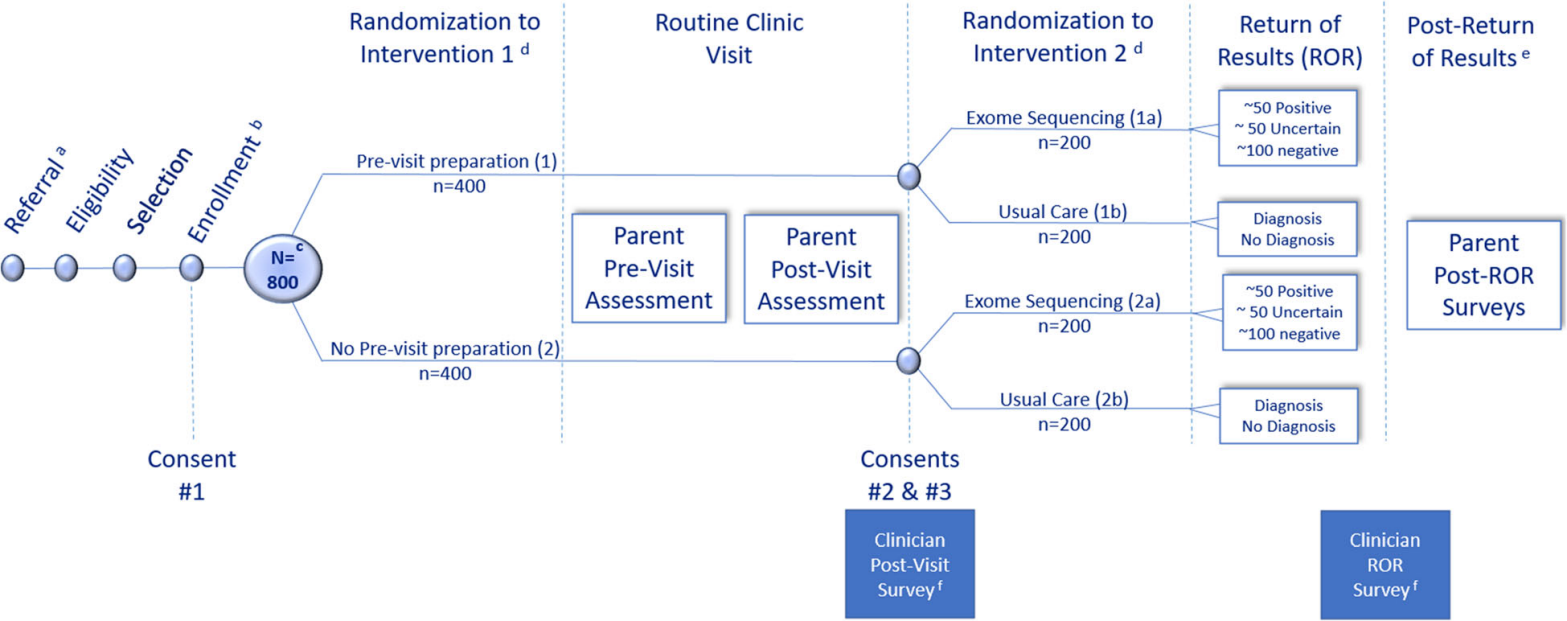
NCGENES 2 study recruitment, enrollment, and clinical trajectory with trial arms and anticipated sample size. ^**a**^All eligible participants are new patients presenting for evaluation to pediatric genetics or pediatric neurology clinics. ^b^Enrollment is completed by phone before the scheduled new patient visit. ^c^Planned enrollment is 850 parent-child dyads, for ease of distribution across groups, 800 was used here. ^d^Intervention 1 (PVP) is a behavioral intervention that involves randomizing parents/guardians to receive or not receive a pre-visit educational booklet and a question prompt list for their child’s first clinic visit. Intervention 2 (ES) is a diagnostic intervention where parent-child dyads are randomized to be offered first-line exome sequencing for the child. The trial applies a full-factorial design, resulting in four arms, as illustrated in the figure: 1a PVP, exome sequencing, and usual care; 1b PVP and usual care; 2a exome sequencing and usual care; and 2b usual care (control arm). Estimates of positive, uncertain, and negative findings in the groups receiving ES are based on prior experience. ^e^Parents complete two post-return of result (ROR) surveys: (1) 2-week post-ROR (approximately 6 months after the clinic visit) and (2) 6-month post-ROR (approximately 12 months after the clinic visit). ^f^Clinicians complete survey measures at two timepoints: (1) post-visit survey after the clinical interaction with the child and parent and (2) approximately 6 months after the clinical visits when clinical diagnostic and ES results (if relevant) have been returned

Parent-child dyads will be randomized to the study arms with all possible combinations of the two interventions (PVP and ES) and usual care: (1) PVP + ES + usual care, (2) PVP + usual care, (3) ES + usual care, and (4) usual care alone (Fig. 1). Once randomized to trial arm, the intervention assignments will not be modified. The study will provide evidence as to whether a theory-based, multi-component PVP intervention helps parents access and understand the information they need to engage more actively in their child’s care. The study’s design will also provide evidence about the effect of early use ES on the diagnostic odyssey for pediatric patients with suspected genetic conditions, regardless of their insurance status or ability to pay.

### Pre-visit preparation

Research has shown that minority and medically underserved patients are less likely to be actively involved in clinical encounters and tend to ask fewer questions [42–46]. Active involvement allows patients to share their concerns and priorities, clarify uncertainties, and obtain new information; thus, this involvement is critical for shared decision making and patient-centered care. In the context of ES for minor patients with suspected genetic diseases, it is also important that patients/parents share information about symptoms that can guide the interpretation of variants. Achieving these objectives can be challenging in minority or medically underserved patients/parents, who may prefer a less active role in decision making [47–49]. However, providing tools to increase health knowledge and encourage participation in informed choice and collaborative decision making, has been shown to improve decision self-efficacy, preference for collaborative decision making, and reduced decisional conflict [50, 51].

One tool to promote active involvement in clinical consultations is the provision of a carefully constructed question prompt list (QPL), a communication aid that provides patients with a list of questions to ask their physicians [52]. Patients who use QPLs ask more questions and elicit more information in clinical consultations [53], especially when physicians endorse the use of the QPL and encourage question asking [54]. Evidence suggests that QPLs may be especially important for promoting active involvement of minority and medically underserved patients [42–46].

The study was designed to evaluate the efficacy of a PVP intervention consisting of two packets containing educational booklets and QPLs mailed at two different times to participants before study-associated clinical interactions that are designed to maximize their engagement in clinic activities, informed decision making about genetic testing, and understanding of genetic results. The first PVP packet will focus on helping patients understand what happens in a specialty clinic consultation (e.g., what questions the physician may ask, patients’ role in asking questions and sharing information) and concepts such as genetic causes of health conditions, the process of diagnostic testing, and implications of finding a genetic cause of a health condition. The second packet will focus on different types of results that may be returned from a genetic evaluation, how results may affect the clinical guidance offered by a provider, next steps (e.g., further testing, clinical appointments, family studies), and when/how to talk about the results with family members. Parent and provider surveys and audio recordings of clinical encounters will be used to measure the impact of PVP on the primary outcomes of engagement of participants in the clinical interaction and their view of the interaction as patient-centered.

### Exome sequencing

Evaluation of diagnostic health technology (e.g., exome sequencing) must consider both the clinical scenario that prompts the use of this technology and the desired outcome to be achieved by its use. Widespread adoption of ES by practitioners and payers requires evaluation of its “safety, efficacy, feasibility, and the indications for use, cost, and cost-effectiveness, as well as social, economic, and ethical consequences, whether intended or unintended” [55]. Therefore, in addition to evaluating the diagnostic capabilities of ES, it is important to explore other health-related outcomes, including diagnostic thinking, therapeutic choice, medical outcomes, and familial/societal impacts. Thus, a broad definition of clinical utility goes beyond considerations of diagnostic yield to also include patient and family consequences as well as defined health outcomes.

The second intervention seeks to provide information about the clinical utility of ES as it is broadly defined by randomizing participants to be offered ES testing versus no offer of ES testing. In addition, the current study will build upon our experience in sample tracking, library preparation, and sequencing and analysis developed during the original NCGENES study.

### Study sample and study selection

Study participants will be recruited from three healthcare facilities in North Carolina that are characterized as either academic or community institutions: the University of North Carolina at Chapel Hill/UNC Health (UNC), Mission Health/Healthcare Centers of America (Mission/HCA), and East Carolina University (ECU). These institutions include catchment areas that span the state from east to west and serve a highly diverse patient population, including significant proportions of medically underserved individuals as well as those from racial or ethnic minority groups that are under-represented in research; both considered groups-of-interest in this study. Each partner site will include either a Pediatric Genetics clinic (Mission/HCA and ECU) or both Pediatric Genetics and Pediatric Neurology clinics (UNC). These clinics provide care to a high proportion of patients with phenotypes caused by heterogeneous genetic conditions. Specific medical doctors, genetic counselors, nurses, and certified medical assistants from each clinic will serve as the study’s clinicians. Patients with first-time visits scheduled in study clinics with study clinicians will comprise the pool of potential study participants.

One study goal is to enroll at least 60% of the NCGENES 2 study participants from medically underserved and/or historically under-represented minority populations. To achieve this goal, we will implement a randomized recruitment sampling method to select potential participants to approach for recruitment. This selection method will allow for oversampling of participants from those populations and will provide a flexible alternative to frequency matching [56]. Potential participants will be individually randomized to be recruited or not based on investigator-imposed, clinic-specific recruitment probabilities determined by the demographic characteristics of the clinics’ patient population by race, ethnicity, and insurance status. In NCGENES 2, pediatric participants will be defined as underserved if they have no health insurance or are covered by a publicly funded children’s health insurance program, such as North Carolina (NC) Medicaid or NC Health Choice. They will be defined as historically under-represented in genomic research if they are non-White or Hispanic. Participants selected for the study will then be evaluated for study eligibility.

### Eligible participants and recruitment

Pediatric patients will be eligible if they are being referred for a first-time appointment scheduled at least 3 weeks away with a physician at a study-affiliated clinic, are younger than 16 years of age at the time eligibility is determined, and have defined clinical symptoms with unknown etiology that may be due to an underlying genetic condition (see additional file 1). A team of physicians and genetic counselors developed and will regularly discuss the clinical criteria guide for eligibility. When a child’s phenotypic eligibility is unclear, a central study physician will adjudicate. The clinical criteria will help identify patients for whom ES may help determine the cause of their condition, and thereby may impact the management of their medical condition. All eligible pediatric patients will need an eligible primary guardian (usually a parent) defined as: authorized to provide legal consent and sign legal documents for the child, able to complete written surveys in English or Spanish, being 18 years or older, and willing and able to attend the study-related clinic visit and complete all study-related tasks. The term “parent” is used in this article to refer broadly to adult guardians or caregivers of the pediatric patients who have the authority to consent the child to research. Parents of eligible pediatric patients will receive a letter informing them about the study and later receive an enrollment phone call where parent eligibility will be determined, and a formal invitation to the study will be provided.

### Consent process

The study will use a multi-staged informed consent process due to the complexity of the study (Table 2); our goal will be to avoid overloading participants with complex information by optimizing the time and resources they can use to understand and carefully consider whether to participate. This approach will allow for the continued establishment of trust and rapport. Consent 1 to participate in the study will occur at enrollment and be conducted by a study coordinator or research assistant by phone. The study coordinator will conduct consents (and assents) 2 and 3, as well as inform parents about the Health Insurance Portability and Accountability Act (HIPAA). Ideally, consents 2 & 3 will be done in person immediately following the post-visit assessment, but, if schedules do not allow, it will be completed later by phone. Parents will provide consent for their parent-child dyad; however, children who are chronologically and developmentally 7 years or older will be asked to assent to both the randomization to offer of ES vs. no ES (at time of consent 2) and to providing a biospecimen sample (by either blood draw or saliva collection) if randomized to the ES intervention arm (at time of consent 3). Developmental age will be determined by the physician who conducted their clinical appointment. When the child can assent, the parent’s consent and the child’s assent must be concordant for consent 2 and consent 3. Of note, consent and assent to randomization to ES (consent 2) must be obtained for continued participation in the study. Participants can withdraw consent (or assent) at any time for any reason. They can also be discontinued from further participation by the investigative team if, after consenting, they are determined to be ineligible, either administratively (e.g., child is identified as a ward of the state/foster care) or due to a change in clinical criteria (the patient is determined to have a genetic diagnosis during the study clinical visit).

**Table 2:** Multi-stage consent and assent process

	Consent step	Coverage	Who is offered consent?	Assent component	Timing
*Intervention 1*^a^	*Consent 1*	- Randomization to PVP (versus not getting PVP) - Intake, pre-visit, and post-visit surveys	Every parent participant	No	During enrollment call by phone
*Intervention 2*^a^	*Consent 2*	- Randomization to be offered ES (versus not being offered ES) - Acknowledge discussion of patients’ rights under the Health Insurance Portability and Accountability Act (HIPAA) - Sharing of child’s and parent’s study data with approved investigators and databanks - Use of the child’s medical record until 18 years of age - Linkage of study data to public and private datasets (e.g., health insurance claims data) - Re-contact - Opt-in or out of future research use of study data	Every parent participant who completes a clinic visit^c^	Yes^b^	After the clinic visit
	*Consent 3*	- ES with clinical confirmation - Results to be placed in the child’s medical record - Future use of biospecimen and associated data - Opt-in or out of medically actionable results unrelated to child’s condition (i.e., secondary results) - Opt-in or out of future use of child’s specimen/DNA and related data	Every parent/child dyad randomized to ES	Yes^b^	

^a^Intervention 1 is the pre-visit preparation intervention, and Intervention 2 is the exome sequencing intervention

^b^Children are determined to be developmentally able to assent if they are both chronologically and developmentally 7 years or older. Developmental age is determined by the physician who conducted their clinical appointment

^c^In rare cases, the child may receive a diagnosis at the clinic visit and be withdrawn from the study and not be offered consent 2

### Stakeholder engagement

An overarching goal of CSER 2 and NCGENES 2 is to ensure the outcomes of genomic medicine research are relevant and the potential benefits accessible to diverse populations that encompass medically underserved and under-represented individuals. To accomplish this goal, we will engage stakeholders in concerted multimodal efforts to gain insight from clinician and parent/caregiver stakeholders that may influence research decisions throughout the project.

Clinician stakeholders from the different research sites in North Carolina were actively involved in the pre-award/grant-development period to outline plans that met study goals and clinic needs. Continued engagement with the clinician stakeholders through regular meetings and communications enabled necessary troubleshooting of clinician workflows and measures, as well as the ability to partner on data analysis and outcome dissemination. Insight from parent/caregiver stakeholders was also essential to the development of participant-oriented processes and materials. We have engaged three parent/caregiver groups: (1) individuals with prior genomic research experience; (2) individuals with personal experience as a caregiver of a child with a genetic condition; and (3) individuals who are caregivers and/or advocates for families of children with neurodevelopmental needs. Parents/caregivers who had participated in the original NCGENES study from under-represented and underserved population groups provided experiential perspective on motivations for research participation, ways to facilitate participation by supporting families through long clinic visits, and ways to encourage engagement between caregivers and their child’s doctors [57]. Recruitment telephone scripts and participant compensation plans were revised with feedback from caregiver members from a sickle cell disease community network. A Community Consult Team (CCT) was established to provide continuing materials refinement and ongoing insight. The CCT comprises a diverse group of individuals who are parents of children with neurodevelopmental needs, and/or advocates for families, especially those with special needs children [57]. Most of the CCT members also identify as members of under-represented and underserved populations. Early engagement with the CCT enabled the integration of revised language and format for consent, educational, and survey materials that are more responsive to the needs of our target study populations. Engaging with diverse stakeholders early and throughout the research process will enable our study to be more responsive to the needs of our diverse population and to troubleshoot recruitment and retention challenges as they arise. Throughout the study, all stakeholders (clinical and non-clinical/community) will continue to be informed of study progress and outcomes through regular meetings and periodic newsletters.

## Trial procedures

### Pre-visit procedures

**Fig. 2:**
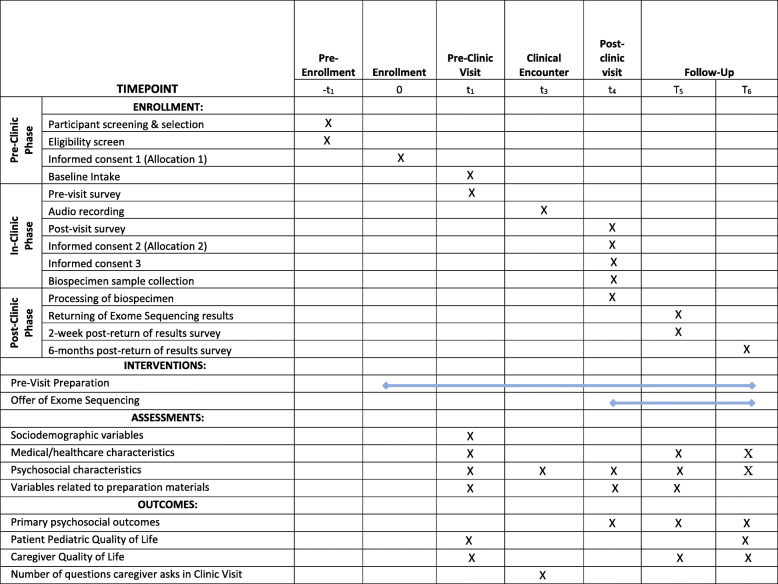
SPIRIT figure. Schedule of enrollment, interventions, and assessments for the North Carolina Clinical Genomic Evaluation of Next-generation Exome Sequencing (NCGENES) 2 randomized controlled trial

After joining the study (consent 1), parents will be sent a packet that is tailored to their assigned PVP study arm. Specifically, parents randomized to receive no PVP (i.e., usual care) will get a reminder letter with information about their research and doctor appointments, an instruction sheet explaining how to go through their packet’s contents, a document reiterating information from consent 1, and a self-administered intake questionnaire specific to the age of the child at the time of enrollment. The intake questionnaire will be designed to take about 30 min to complete and will collect baseline data, including measures of sociodemographic, medical/healthcare, and psychosocial characteristics (see details in Table 3). Age specificity of the intake questionnaire will allow study staff to obtain an age-appropriate pediatric quality of life measure. Parents randomized to the PVP study arm will receive the same materials as well as a pre-visit educational booklet and QPL. When possible, the study staff will give parents at least 14 days to review and complete the contents of the packet before their child’s clinic visit Fig. 2.

**Table 3:** Parent measures and assessments

		Assessments				
Measure	Number of items	***Intake***	***Pre-clinic visit***	***Post-clinic visit***	***Two-week post-return of results***	***Six-month post-return of results***
Sociodemographic variables^a^		X				
**Medical/healthcare characteristics**						
Length of diagnostic odyssey ^c^	1 item	X				
Access to care ^a, c^	2 items	X				
Pediatric quality of life [PEDS-QL] [58]^a^	23 items	X				X
Parent quality of life [SF-12] [59]^a^	12 items		X		X	X
Perceived health of caregiver^a^	1 item		X		X	X
Perceived health of child [60]^a^	1 item		X		X	X
**Psychosocial characteristics**						
Preferred control in medical decisions [61]	1 item	X				X
Decision self-efficacy [62]	7 items	X	X		X	X
Missed work due to child’s condition [63]^b^	1 item	X			X	X
Financial toxicity [64]	11 items	X			X	X
Group-based medical mistrust [65]	12 items	X				X
Personal physician trust/mistrust [66, 67]	8 items	X				X
Genomic knowledge [UNC-GKS] [29]	25 items	X				X
Health literacy [BRIEF] [68]^a^	4 items	X				
Subjective numeracy [69]^	3 items	X				
Depressive symptoms [PHQ-8] [70]	8 items		X		X	X
Anxiety [GAD-7] [71]	7 items		X		X	X
Emotional state [PANAS] [72]^b^	22 items			X		
**Variables related to preparation materials**						
How much of the prep materials did caregiver review^c^	1 item		X		X	
Helpfulness of prep materials ^a^	Varies by time point		X	X	X	
**Primary psychosocial outcomes**						
Perceived patient-centeredness of clinical consultation [73]	21 items			X	X	
Number of questions asked during clinic visit	Coded from audio recordings			X		
**Variables related to diagnostic testing & return of results**						
Understanding of diagnostic testing [74]^c,b^	6 items Checklist items vary by timepoint			X	X	X
Patient experience and satisfaction [75]^b^	Varies by time point			X	X	
Understanding of diagnostic testing results^a,d^	1 item				X	
Reaction to diagnostic testing results ^c^	1 item				X	
Trust in results ^c^	1 item				X	X
Feelings about genomic testing results [FACToR] [76]^a^	14 items Scale				X	X
Personal utility of diagnostic results [PrU] [77] ^a^	17 items				X	X
Self-efficacy for explaining diagnostic results ^c^	5 items				X	
Assessment of communication effectiveness [78]^a,b^	15 items				X	

^a^CSER-harmonized measures

^b^Adapted measure

^c^Developed for NCGENES 2

^d^Developed for CSER 2

### In-clinic procedures

When families arrive for the child’s clinic visit, a research assistant will collect their completed study intake questionnaire and provide parents with a tablet computer to complete the pre-visit questionnaire. This assessment will be accessed via a personalized link, and participants’ responses will be entered directly into the study’s web-based patient tracking system. Measures that will be included in the pre-visit questionnaire are shown in Table 3. For participants randomized to the PVP intervention, the web-based patient tracking system will ensure that the intake questionnaire will include items assessing whether parents reviewed the PVP materials and ask parents to rate the helpfulness of the materials; the research assistant will be blind to participants’ study arm and thus will not know whether these items are administered.

After completing the pre-visit questionnaire, parents will have the option to provide their permission to audio record the clinic visit. The audio recordings will allow for qualitative analysis of clinician-patient interactions, as required for our primary outcome (e.g., the questions parents asked during the clinical consultation and how the providers responded to the parent). Regardless of a parent’s decision to allow audio recording, all dyads will proceed to have a usual care consultation with the child’s clinical team. Following the consultation, parents will complete the post-visit questionnaire (via the study tablet), which is also accessed via a personalized link allowing parents to enter responses to measures directly into the study’s web-based patient tracking system. The post-visit questionnaire measures are shown in Table 3. For parents assigned to the PVP intervention, the tracking system will ensure that this assessment includes items evaluating the helpfulness of the PVP materials during the visit with the physician. As with the pre-visit questionnaire, the research assistant will not be aware of the administration of these survey items.

After completing their post-visit survey, parents will meet with the study coordinator to discuss the HIPAA, the consent (and, if necessary, the child assent) to randomization to ES (Consent 2), and, subsequently, for those randomized to the intervention, the consent to ES (Consent 3). Consent form 2 will inform the parent of the remaining study activities: completion of follow-up surveys, and data collection via review of their child’s medical and insurance records until their child turns 18 (Table 2). Consent form 3 will inform the parent of ES, sample collection, and the type and meaning of the ES results (i.e., positive, negative, of variant of unknown significance) (Table 2). After consent (and if necessary, child assent) is obtained, the study coordinator will provide compensation and, if necessary, escort the parent and child to a phlebotomy station, where two blood samples will be obtained from the child participant and subsequently distributed to the study laboratories. If a blood sample cannot be collected from the child, two saliva samples will be collected by sending kits via mail with collection instructions, and a pre-paid self-addressed return mailing package will be provided to the parent.

### Post-clinic procedures

#### Post-clinic visit physician activities

Study physicians will complete a post-visit questionnaire that assesses medical/healthcare and behavioral characteristics of parent and child participants. Medical/healthcare variables will include the following: children’s phenotypic category, suspected genetic etiology, the physician’s expectations for diagnostic testing (i.e., whether they are ordering diagnostic tests and whether they think that ES would be useful in the diagnostic work-up of the specific patient), whether the physician communicated a next step to diagnosis or recommendations for symptom management, planned medical actions, and the length of the visit compared to average. They will also report how many questions the parent asked, the impact of the parent’s questions on the flow of the appointment, and if they felt the parents were prepared for the appointment. Additionally, physicians will report the patient phenotype using PhenoTips® (a registered trademark of Gene42 Inc. Copyright© 2015–2020 Gene42 Inc) software package. This software will facilitate phenotypic descriptions using a structured set of terms within the Human Phenotype Ontology (HPO) [79].

#### Post-clinic visit, pre-return of results mailing

Approximately 5 months after their child’s clinic visit, parents will be mailed a second study packet. Parents who will receive both the PVP and ES interventions will be mailed a packet that contains an educational booklet and QPL similar to the pre-visit versions of these preparation materials. However, the new content will focus on the return of results and the questions families may have about the ES results they will receive for their child. The educational materials will not be relevant for parents in the PVP arm whose child did not receive ES in the study. Therefore, parents will not be mailed these educational materials if they were randomized to any of the three other study arms (PVP + usual care, ES + usual care, or usual care alone), or if they were randomized to receive ES but decline sequencing. However, parents in these other study arms will receive a letter reminding them that they will be receiving a follow-up survey asking about any clinical results that were returned for their child.

### Biospecimen distribution, processing, analysis, and results reporting

Once obtained, paired blood or saliva specimens (identified only with the unique study identifier) will be distributed to the study labs. For each participant receiving ES, one sample will be distributed to the UNC Biospecimen Processing (BSP) research laboratory and a second independent sample to the CLIA-certified UNC McLendon Molecular Genetics Laboratory (MGL).

DNA samples to be used for research ES will be isolated and aliquoted in the BSP. Sequencing libraries will be prepared with Agilent SureSelect XT (Human All Exon V7) using the manufacturer’s low input protocol guidelines. Sequencing will be performed in the UNC’s High-Throughput Sequencing Facility (HTSF) on an Illumina HiSeq4000 with a minimum average target depth of 50×. Raw sequence reads will be mapped using BWA-MEM [80] version 0.7.17, duplicate reads will be marked with Picard MarkDuplicates version 2.18.14, and variants called using FreeBayes [81] version 1.3.1. Bioinformatic processing will include annotation and prioritization according to previous variant interpretation assertions based on ClinVar entries, minor allele frequency in reference databases such as GnomAD [82–84], and predicted effect of the variant on the protein.

Molecular analysts will review all rare potentially damaging (i.e., truncating (nonsense, frameshift), splice site, and missense) variants in gene lists from Genomics England PanelApp [85], which will be selected by a study clinical geneticist to match with participant phenotypes as provided by the study clinicians. Variants will be classified according to guidelines from the American College of Medical Genetics and Genomics and the Association for Molecular Pathology [86] with updated recommendations from the Clinical Genome Resource (ClinGen) consortium [86–88]. The primary analyst will flag variants classified as “pathogenic,” “likely pathogenic,” or “variant of uncertain significance” in genes associated with conditions relevant to patient phenotype, or classified as “pathogenic” or “likely pathogenic” in genes associated with actionable secondary findings for review by a committee of molecular geneticists, physicians, genetic counselors, and researchers. This committee will meet weekly to discuss variants for further review and to make consensus decisions about which variants will be confirmed (and if so, reported) in the MGL CLIA-certified lab.

Samples received by the MGL will undergo DNA extraction independently from the samples processed in the research pipeline and will undergo genotyping of a pre-established set of single-nucleotide variants to compare with research sequencing data for identity confirmation. The DNA samples will be subsequently held for potential clinical confirmatory testing of variants identified via the molecular analysis committee. ES findings confirmed in the MGL and determined to be explanatory, possibly explanatory, or returnable secondary findings [89] will be placed in the child participant’s electronic medical record with automated physician notification. The findings will also be sent by the study team to the study clinicians via automated email notification and made available in the study’s web-based patient tracking system. Negative research ES results will not be included in the medical record and instead will be reported to the clinicians only via the study team by automated email notifications and the study’s web-based patient tracking system.

If the study physician deems it necessary to obtain genetic testing on parents or other pertinent relatives to interpret the ES results of the child (e.g., to determine phase or identify whether a variant is de novo), then relatives will be consented for variant-targeted sequencing by phone. Prior to phone consent, relatives will receive the consent form by mail along with a pre-paid self-addressed envelope to return the signed consent to the study team. After the signed consent form is received, saliva kits will be mailed to the relative and returned to the study team for distribution to the clinical lab. Genetic test results for the parent or other relative will be added as an addendum to the child’s clinical molecular genetics report within the electronic medical record. The child’s result, including results amended based on family testing, will be communicated to the family by the clinical team. Detailed protocols for laboratory methods will be available upon request.

### Post-return of results activities for parents and clinicians

Results will be disclosed to parents by the clinical team according to usual care practices. Most frequently, results will be returned to the parent by phone, either by the physician, a genetic counselor, or both. Results reporting will rarely require a return visit to the clinic. After results are returned, physicians will complete a second study questionnaire that will include measures of their confidence that a primary causal etiology was identified, the likelihood the patient has a genetic condition, diagnostic outcomes, planned medical actions, the content of the return of results discussion, whether they ordered testing for other family members, the diagnostic utility of the result, and the length of the consultation compared to average. Physicians will answer these questions about the main diagnostic test completed for their patient, which would be ES for parent-child dyads randomly assigned to that intervention (unless the parent or child declined it) or a different type of diagnostic test for other patients. If patients do not undergo diagnostic testing, physicians will not be presented with questions relating to diagnostic testing and results. Providers (physicians and genetic counselors) involved in disclosing results will also complete measures about perceptions of patient engagement, perception of family’s access to resources, and provider confidence in parents’ ability to understand, explain, and manage care based on the result.

For parent-child dyads assigned to have ES, a return of results questionnaire will be sent approximately 2 weeks after the child’s ES results are returned (triggered by the clinician’s completion of the clinician’s return of results questionnaire). While the turnaround time for research ES may be variable due to the need to batch samples, it is expected to be approximately 6 months after the child’s clinic visit. A research assistant will mail the parent participant a return of results questionnaire with instructions for completing and returning it via mail. For parent-child dyads not assigned to ES or for those refusing ES, a return of results questionnaire will be sent approximately 6 months after the child’s clinic visit to capture any result disclosures that may occur due to other tests ordered by the physician. In addition, parents will complete a final follow-up questionnaire 6 months after their return of results questionnaire (approximately 1 year after their child’s clinic visit). Beyond these questionnaires, the study team will continue to monitor the child participant’s medical record and insurance claims data for patterns of health care until age 18. Additionally, a child participant’s vital status will be monitored monthly though the North Carolina Department of Health and Human Services Vital Statistics database.

### Study retention and minimizing burden

The NCGENES 2 study will employ several methods to minimize potential study burden and maximize retention of participants throughout the study. Specifically, during the child’s clinic visit, a research assistant will greet the families upon arrival and assist with their navigation throughout the clinic visit. Research assistants will also provide snacks for the parents and children, as well as a tablet computer with movies and cartoons for the children to use while their parents complete the pre- and post-visit questionnaires. The study coordinator will also give children a small gift as a token of appreciation for their participation and provide parents a cash compensation for their travel costs and time. After this visit, research assistants and study coordinators will mail birthday cards to child participants and “thank you” notes 3 and 9 months post-enrollment to parent participants. Parents will complete hardcopy intake forms and follow-up questionnaires at home at their convenience, which should lessen the potential burden on them (e.g., compared to online assessments or scheduled phone interviews). Parents will return these materials in an addressed and pre-paid postage return envelopes. Additionally, parents will receive “thank you” notes with gift cards after returning each follow-up questionnaire (approximately 6 and 12 months post-enrollment). Thus, parents and children will be contacted at 3-month intervals throughout the study to encourage continued study engagement and hopefully ensure the study maintains current contact information. The study’s CCT may help highlight other potential barriers to recruitment and retention and offer suggestions [57].

### Randomization and concealment

Participants will be randomized in a 1:1:1:1 allocation ratio to the study arms with all possible combinations of the two interventions (PVP and ES) and usual care. The rationale for randomization is to directly compare outcomes in patients who receive pre-visit preparation to those who do not, and who undergo ES at an early point in the diagnostic process to those who have usual care. Random assignments will be concealed electronically until the time of disclosure using an automated web-based patient tracking system. Additionally, the tracking system will limit access to the randomization status information by study role and by the participants’ status along the study trajectory. Therefore, only staff in roles that need this information will be able to access it, and information will only be accessible when it is relevant for the study participant, given their status in the study trajectory.

Random assignment to the PVP intervention will be revealed to and by the study coordinator after the parent consent to participate in the study. Other study staff (e.g., research assistants and clinicians) will remain blind to the participants’ PVP intervention status. However, study clinicians may become unblinded to the participant’s PVP random assignment during the clinic visit. For example, unblinding of the clinician could occur when participating parents use the QPL appropriately during the clinical consultation to facilitate patient-clinician communication. They may also bring the PVP educational booklet to the visit; thus, if parents use the pre-visit booklet and/or QPL during the clinical visit, the clinician will know (appropriately) that the parent was assigned to the PVP study arm.

Randomization to the ES intervention will be determined simultaneously with the PVP intervention. However, this information will be concealed from study staff, clinicians, and participants until the parent consents to randomization to ES after the clinical visit (for the study coordinator, parent, and child) and after the clinician completes their post-visit survey. All concealments are strategically placed to avoid biasing staff or clinicians’ interactions with the participants or participant retention based on study arm assignment. Further, parents will complete the 2-week and 6-month post-return of result follow-up measures without assistance from study staff, eliminating the potential for bias in the follow-up assessments.

For any data analysis, investigators will receive coded data without identifiers and thus will not be able to link a patient’s name to an intervention arm. Some analyses may require individual-level randomization data to allow for comparisons, for example, of parent questionnaire responses or health outcomes by trial arm—a major focus of this study. Thus, in several evaluations, the analyst will not be blind to the participant’s intervention status.

### Data monitoring

We will ensure data quality throughout the study with extensive staff training, monitoring, and availability of a detailed protocol. A web-based participant tracking system developed specifically for the NCGENES 2 study will facilitate data monitoring. The tracking system also will enable the secure collection, storage, and management of paper documents, electronically signed consent and assent forms, participant data, lab reports, and audio files. The tracking system’s logic checks and requirement for in-range variable responses will optimize data integrity. Additionally, an experienced biostatistician will perform weekly to monthly monitoring of study activities and data collection quality and produce reports of missing values and inconsistent study events for resolution. Cross-site, bi-annual monitoring visits will be done. At these visits, data quality and protocol maintenance will be evaluated, after which staff members may be retrained and recertified to assure data and study integrity.

### Measures

#### Parent measures

Parents or primary guardians of pediatric patients will complete measures at five time points: (1) after study enrollment (intake questionnaire), (2) in the clinic before their appointment (pre-visit questionnaire), (3) immediately after their appointment (post-visit questionnaire), (4) approximately 6 months after their child’s initial clinic visit (2-week post-return of results questionnaire), and (5) approximately 12 months after the child’s initial clinic visit (6-month post-return of results questionnaire). The last two time points are follow-up periods after the parents received their child’s ES or other clinical test results. Parent measures are listed in Table 3, which also identified the measures that have been harmonized across CSER 2 sites to leverage additional sample size and enable cross-site analyses and comparisons. When possible, we selected measures that have been shown to have good reliability and validity in prior research and that are appropriate for diverse patient populations. Some measures were developed for NCGENES 2 or the CSER 2 consortium, with guidance from content experts and experts in measurement and psychometrics.

The primary psychosocial outcome variables are parents’ perception of the patient-centeredness of the clinical consultation and coded communication behaviors (e.g., the number of questions parents asked during the appointment) from transcriptions of audio recorded at a child participants’ clinic visit [73]. Codes are being developed after initial readings of the transcripts and adapted from prior studies [90–93].

Measures developed for CSER 2 and NCGENES 2 will allow study staff to evaluate participants’ expectations of and experiences with diagnostic ES. The goal in selecting constructs to measure was to assess parents’ experience of their child’s genomic sequencing, their understanding of sequencing and sequencing results, and their behaviors and psychosocial outcomes after receiving their child’s results. These measures are summarized in Table 3.

#### Clinician measures

Sociodemographic and professional characteristics (e.g., provider’s role, specialty, genetics education, years in practice, and race and ethnicity) will be collected for the study clinicians. Also, clinicians from the pediatric clinics will complete study measures for each participating dyad at two time points: immediately after the appointment (post-clinic visit), and 6 months after the child’s clinic visit or 2 weeks after exome sequencing results are returned to the patient’s parent (2-week post-return of results). Clinicians will be alerted of the questionnaires via email and will complete them on their computers. All clinician measures used in this study were developed by the CSER 2 Measures and Outcomes Working Group or the NCGENES 2 study team.

### Planned analyses

Analyses will begin with descriptive statistics for all study variables. The distribution of continuous variables will be assessed, and normalizing or variance stabilizing transformations will be applied where necessary before conducting further analyses. Current methods for evaluating patterns of missing data and for imputing missing values will be used when appropriate. We will evaluate the success of randomization (i.e., to ensure that participants in the study arms do not differ on demographic or other relevant variables). Variables shown to be significantly different between the groups will be included as covariates in multivariate models. An intention-to-treat approach will be used to compare groups. The ultimate analytic approach for the analyses will be determined by the nature of the outcomes (discrete or continuous), the need to include covariates, and the research question being addressed.

We will explore site-study arm interaction to evaluate whether the effects of trial arm assignment differ across sites. Further, analytic adjustments will be applied where necessary for clustering by the dyad, site, or clinic and will account for randomized recruitment sampling. Adjustment for multiple comparisons between arms will be considered, if necessary. Bonferroni or Tukey corrections will be used to control the overall type-I error rate. An adjusted p value smaller than 0.05 will be considered statistically significant. Analyses will be performed by an experienced biostatistician and will apply currently recommended approaches used in randomized controlled trials and subgroup analyses [94].

An outline of the primary and secondary outcome measures of interest can be found on the NCGENES 2 ClinicalTrials.gov page [95]. One primary outcome of interest, patient-centeredness, will be assessed in the post-visit and 6-month post-return of results follow-up surveys. We expect to see an effect of study arm hypothesizing that participants who received PVP will perceive their physician’s care to be more patient-centered and ask a greater number of questions than participants who did not receive pre-visit education. We will explore the effects of random assignment to ES + usual care vs. usual care alone, and the joint (interactive) effects of the ES and PVP interventions. Changes in effects over time will also be evaluated. Secondary outcomes that are measured longitudinally will be analyzed similarly to the primary outcomes.

### Sample size

NCGENES 2 seeks to enroll 850 parent-child dyads (1700 total participants) into the study. We anticipate approximately equal numbers of participants in each of the four intervention groups. Power analyses were conducted to evaluate statistical power for evaluating the primary quantitative outcomes. Evidence shows that the standard deviation of perceived patient-centeredness of a clinical consultation ranges from 0.8 to 1.3 [96], and the standard deviation of the number of questions patients ask during a clinical consultation ranges from 2.89 to 7.22 [54, 97]. The anticipated intervention effect size measured in the difference between any two of the four arms is more than 2. Hence, conservatively, our sample size as a 1:1 ratio (425 versus 425) has 100% statistical power to detect such a difference, even under a Bonferroni correction for multiple comparisons between four arms, i.e., under 0.05/6 = 0.008 type-I error rate. We also have sufficient power to explore multiple regression models with two covariates of substantive interest (underserved and under-represented statuses) and eight covariates (e.g., demographics). Using 850 outcomes, we would have 90% power in testing the significance of the two covariates of interest when they explain only 1.5% of the variance not explained by the other eight covariates.

### Ethical and regulatory considerations

NCGENES 2 is a registered clinical trial [ClinicalTrials.gov Identifier: NCT03548779]. The study’s use of ES was determined by the UNC IRB to be an exempt diagnostic device that (1) is non-invasive, (2) does not require invasive sampling that presents significant risk, (3) does not introduce energy (e.g., surgical lasers) into the subjects, and (4) the results from which will not be used to diagnose or make treatment decisions without confirmation using a medically established test (e.g., clinical confirmation in a CLIA-approved diagnostic laboratory). Given the minimal risk determination, the UNC IRB provides central regulatory oversight for the NCGENES 2 study and therefore has established reliance agreements with both partner sites at ECU and Mission Health/HCA. Additionally, each partner site has an established data use agreement with UNC to facilitate study implementation and review. UNC will monitor partner study sites, and partner sites will monitor UNC at least bi-annually for study documentation, study protocol compliance, quality integrity of the data collected, and adherence to good clinical practices. Monitor visits will be followed by a debriefing session with staff where concerns will be addressed and timing and plans for remediation (e.g., retraining) will be documented and agreed upon by the study team and monitor.

Study sites will adhere to established patient confidentiality and privacy training, and requirements and will secure data in compliance with robust institutional information security programs. As a study funded by the National Institutes of Health (NIH), NCGENES 2 will assure submission of study data to an NIH-designated data repository that meets expectations defined by the Genomic Data Sharing Policy. Those expectations require that the identities of research participants will not be disclosed to NIH-designated data repositories, an IRB has reviewed the investigator’s proposal for data submission and assured that the protocol for the collection of genomic and phenotypic data is consistent with Code of Federal Regulations, Title 45 (Public Welfare), Department of Health and Human Services, Part 46 (Protection of Human Subjects (45 CFR Part 46), and that data submission and subsequent sharing for research are consistent with the informed consent signed by study participants. As a study enrolling pediatric patients, many of whom also have developmental and physical disabilities, NCGENES 2 will prioritize the protection of this vulnerable population by ensuring ethical research conduct. Specifically, in accordance with the Standard Operating Procedures of the UNC Office of Human Research Ethics, NCGENES 2 requires concordance between parent consent and child assent, particularly when collecting written assent for children who are chronologically and developmentally 7 years of age or older (see “Consent process”).

The NCGENES 2 study will be internally monitored by a steering committee with expertise in clinical and molecular genetics, genetic counseling, social/health psychology, health economics and policy, epidemiology, project and regulatory management, and biostatistics. The committee will be comprised of the study’s principal investigators, study staff, and working group leadership. Additionally, the NCGENES 2 steering committee will serve as the primary body for managing and vetting presentations and publications (NCGENES 2-specific and collaborations across the CSER consortium). This committee will help manage authorship assignment, ensure feasibility of the concepts proposed, and assess manuscripts for overlap. Disputes regarding authorship will be adjudicated by the committee’s chair.

### Potential harms and patient safety monitoring

Patient-facing study protocols have been developed according to the ethical principles of confidentiality, privacy, and beneficence. The clinical team will regularly monitor and report to the NCGENES 2 Steering Committee any safety concerns and all adverse events within 24 h, in addition to commencing the necessary action to resolve them. The study team will adhere to a rigorous safety plan and monitor any instances of suspected study-related harms—including both self-monitoring by site and across-site monitoring. All potential patient safety or adverse events and their resolutions will be documented and appropriately reported (e.g., to IRB) during the study period. For example, because parental anxiety and depressive symptoms will be assessed in the study, we will have an established protocol for contacting parent participants who report clinically elevated anxiety and depression symptoms, as indicated by their scores on validated study measures to ensure that they have resources to cope. The protocol will include a free phone consultation by a study team clinical psychologist. When acceptable to the parent, the psychologist will provide a referral to local mental health and related resources.

## Discussion

The NCGENES 2 study is being led by an interdisciplinary team implementing a multi-site randomized controlled trial focused broadly on understanding the clinical utility of early ES in pediatric patients that include populations historically under-represented in research and typically underserved medically. At the time of this paper, enrollment is ongoing and typically begins 5 to 6 weeks in advance of clinic visits. At UNC, enrollment began on August 28, 2018 (with the first clinic visit October 3, 2018), at Mission/HCA on September 13, 2019 (with the first clinic visit October 28, 2019), and at ECU on February 17, 2020 (targeting an April 2020 start of clinic visit). Due to the COVID-19 pandemic in mid-March 2020, the start of in-person clinic visits at ECU targeted for April 2020 was postponed, and enrollment activities at all sites were suspended temporarily but later resumed at all sites, specifically with ECU enrollment resuming on August 17, 2020 (with the first clinic visit September 21, 2020). Additionally, at the time of this paper, study documents are only available in English, and Spanish translation is underway. Spanish-speaking study staff have been hired and trained and study document translation is underway. Once translation is complete and documents are IRB-approved, enrollment of Spanish-speaking participants will begin at UNC towards the end of 2020.

Employment of genomic sequencing without adequate research involving under-represented and underserved groups could limit the potential benefits of research outcomes and heighten rather than reduce disparities. Thus, evaluating the clinical utility of early ES in diverse populations has specific implications for health equity, particularly in the area of genetic medicine, given persisting disparities and barriers to health care access among underserved and under-represented populations. This study examines the technical, clinical, and health economic issues associated with the utility of early ES technology for shortening the diagnostic odyssey of a diverse cohort of pediatric patients with likely genetic conditions.

Applying the rigorous Phase III randomized controlled trial design in a real-time, real-world clinical setting presents some challenges, and these challenges are amplified in a multi-site study. Recruitment for the study relies closely on the examination of clinical scheduling data. As such, study recruitment is sensitive to the unique and dynamic scheduling characteristics of the different clinics participating in the study. Further, critical components of the NCGENES 2 study (e.g., pre-visit and post-visits procedures and audio recording of clinical visits) are built around routine clinical processes and interaction. Thus, study procedures needed to be developed to allow for the seamless integration of study processes into these clinic visits and processes across the three separate health systems. Given the clinical integration aspect of the study, the COVID-19 pandemic presented a unique challenge to an already complex design and required adjustments to be made across the study sites. For example, the restart of study activities at UNC and ECU required the process for consent forms 2 and 3 to occur virtually (i.e., either by WebEx, Zoom, or teleconference). Hence, prior to a virtual visit, the study team mails all consent and, if necessary, assent forms to the parent to be discussed during the virtual visit. And, with IRB approval, assent procedures have been adapted for families attending virtual clinic visits. Instead, parents are provided the assent form for ES to help them answer questions their child may have regarding this part of the study. If necessary, the parent can call the study toll-free number to have the study coordinator answer the child’s questions. Early and continued engagement of important and diverse stakeholders through the development and implementation phases of the trial has been crucial for developing sensitive approaches to address myriad potential barriers to research processes and participation [57].

Findings from this trial will provide knowledge about the potential benefits of first-line ES when added to routine medical care for specific types of pediatric cases, particularly about the potential to shorten the diagnostic odyssey for pediatric patients and their families. Thorough communication between families and clinicians is a critical component for optimizing successful ES testing. As demonstrated by prior research, implementation of the pre-visit preparation intervention may improve communication and increase shared decision making and engagement in clinical care, particularly for diverse cohorts of patients [43, 45, 51–53]. Thus, findings from this trial will also evaluate the benefits of providing parents with PVP to improve parents’ engagement in their child’s clinical care and to encourage more effective parent-clinician communication. Overall, results from NCGENES 2 and other CSER 2 projects will inform efforts to engage diverse populations in genomic medicine research and also generate evidence that can be used in evaluating the utility of genome-scale sequencing in health care.

## Supplementary Information

**Additional file 1:** NCGENES 2 Clinical Criteria. Clinical screening criteria to determine pediatric patient NCGENES 2 eligibility. NOTE: Criteria evolve as new conditions and symptoms are identified in eligible patients through referral reason and medical record chart review as needed.

## Data Availability

Variant interpretations (not associated with specific cases) will be submitted to ClinVar. Sequence variant and survey data will be submitted to the controlled-access NHGRI Genomic Data Science Analysis, Visualization, and Informatics Lab-space (AnVIL) database. Survey data will be aggregated with CSER consortium data using harmonized measures. Additional trial data and materials will be made available upon reasonable request.

## Abbreviations

BSP: UNC BioSpecimen Processing Laboratory
CCT: Community Consult Team
CHIP: Children’s Health Insurance Plan
CLIA: Clinical Laboratory Improvement Amendments
COVID-19: Severe acute respiratory syndrome coronavirus 2
CSER: Clinical Sequencing Exploratory Research Consortium
CSER 2: Clinical Sequencing Evidence-Generating Research Consortium
DNA: Deoxyribonucleic acid
ECU: Eastern Carolina University
ELSI: Ethical, Legal, and Social Implications
ES: Exome sequencing
GC: Genetic counselor
GS: Genome-scale sequencing
HTSF: High-Throughput Sequencing Facility
HIPAA: Health Insurance Portability and Accountability Act
IRB: Institutional Review Board
Mission/HCA: Mission Health/Healthcare Centers of America
NC: North Carolina
NCGENES 2: North Carolina Clinical Genomic Evaluation by Next-generation Exome Sequencing 2
NCI: National Cancer Institute
NIH: National Institutes of Health
NIMHD: National Institute of Minority Health and Health Disparity
NHGRI: National Human Genome Research Institute
PI: Principal investigators
PVP: Pre-visit preparation
QPL: Question prompt list
UNC: University of North Carolina at Chapel Hill
