# Supplementary material for: Evaluating the clinical utility of early exome sequencing in diverse pediatric outpatient populations in the North Carolina Clinical Genomic Evaluation of Next-generation Exome Sequencing (NCGENES) 2 study: a randomized controlled trial
Source: Trials. 2021 Jun 14;22:395. doi: 10.1186/s13063-021-05341-2 (PMC8201439; doi:10.1186/s13063-021-05341-2)
Supplement: Supplementary file 1 — Additional file 1: NCGENES 2 Clinical Criteria. Clinical screening criteria to determine pediatric patient NCGENES 2 eligibility. NOTE: Criteria evolve as new conditions and symptoms are identified in eligible patients through referral reason and medical record chart review as needed. [file 13063_2021_5341_MOESM1_ESM.pdf]

NCGENES 2 CLINICAL CRITERIA

Clinical screening criteria to determine pediatric patient NCGENES 2 eligibility. NOTE: Criteria evolve as new conditions and symptoms are identified in eligible patients through referral reason and medical record chart review as needed.

Phenotypic Eligibility Criteria

1. **Inclusion Criteria**
    a. Epilepsy and seizures (Any seizures if with developmental delays. Any seizures onset prior to 6 months old. But not for example febrile seizures or a single afebrile seizure in a child with typical development).
    b. Neuromuscular disorder (this may include muscle weakness, elevated CKs, neuropathies, arthrogryposis, etc)
    c. Brain malformation (this includes lissencephaly, schizencephaply, megalencephaly, polymicrogyria, pachygyria)
    d. Intellectual disability/autism spectrum disorder with intellectual disability/developmental delay
    e. Inborn errors of metabolism suspected
    f. Movement disorder (this includes for example dystonia, ataxia)
    g. Microcephaly/Macrocephaly with developmental delays or any that are >2.5 SD from normal.
    h. Some metabolic indications that WILL be included:
        i. R/O mitochondrial disorder
        ii. R/O unexplained metabolic conditions with neurologic component like:
            1. Hypotonia,
            2. Ketosis
            3. Hypoglycemia
            4. Hepatomegaly
            5. Cyclic vomiting
            6. Splenomegaly
    i. Some chromosome Abnormality (some select cases see below)
        i. A microarray VUS would still effectively be in a "diagnostic odyssey" and be eligible, however it could also be a real "positive" in which case the referral would be for post-test counseling and the case would be ineligible.
    j. Hearing loss
    k. Dysmorphic features
    l. Skeletal Dysplasia (e.g., achondroplasia, OI, Unknown)
    m. Multiple congenital anomalies
        i. Includes multiple congenital anomaly syndromes with no etiology (e.g., VATER, CHARGE (make sure not related to CHD 7 gene mutation), Pierre Robin)
    n. Rhabdomyolysis
    o. Hereditary/Childhood Cancer while typically out of scope for NCGENES 2, should not be an absolute exclusion criterion. There are some multisystemic genetic syndromes where cancer can be seen (e.g., Wilms tumor in the setting of overgrowth, neonatal hypoglycemia, and exomphalos would suggest Beckwith-Wiedemann syndrome). When such a condition is described seek study geneticist review and decision.
    p. Other conditions that are considered to have a genetic etiology (such as severe congenital central hypoventilation syndrome).
    q. Fetal Alcohol Syndrome – while an entirely non-genetic disorder it not infrequently can be confused with other genetic syndromes. Specifically, if *developmental delay* is included as a

finding in the child with FAS referral we will consider them eligible. Other cases will be reviewed by the study geneticist for inclusion on a case-by-case basis.

2. **Exclusion criteria**
   a. Patients with known non-genetic causes for child's symptoms (For example; history of the following acquired conditions such as ischemic encephalopathy (including perinatal stroke), CNS infection (such as meningitis or encephalitis), brain trauma, autoimmune/inflammatory disorder or other acquired injury to brain/nerve/muscle. Macrocephaly in a patient with known hydrocephalus/shunt)
   b. Patients who have a known genetic diagnosis that explains the clinical presentation.
      i. Trisomy 21, 18, 17, 16p
      ii. Microdeletion 22q
      iii. Family history of known genetic condition and coming for genetic counseling (e.g., Cystic Fibrosis)
   c. Most metabolic conditions will NOT be included (e.g., abnormal newborn screen)
   d. Isolated findings including but not limited to:
      i. Café au lait (isolated)
      ii. Short stature (isolated)
      iii. Obesity (isolated)
      iv. Failure to thrive (isolated)
      v. Cleft lip and/or cleft palate (isolated - not part of a syndrome)
      vi. Congenital nevus (isolated)
      vii. Congenital scoliosis (isolated)
      viii. Isolated Attention Deficit/Hyperactive Disorder (ADHD)
   e. R/O connective tissue disorder/hypermobility
      i. R/O Ehlers-Danlos Syndrome
      ii. R/O Marfan Syndrome
